# A novel *Arabidopsis* marker line that strongly labels uninucleate microspores and the subsequent male gametophyte development stages

**DOI:** 10.1186/2193-1801-2-237

**Published:** 2013-05-24

**Authors:** José António da Costa-Nunes

**Affiliations:** CBAA - Instituto Superior de Agronomia, Universidade Técnica de Lisboa, Tapada da Ajuda, Lisboa, P-1349-017 Portugal

**Keywords:** Microspore, Pollen, GUS, GFP, At5g17340, Marker line

## Abstract

Here is reported a novel histological marker line that strongly and specifically labels the male gametophyte in *Arabidopsis* flowers, from the uninucleate microspore to the pollination stage. In this marker line, the expression of the *UidA* and *GFP* marker genes is driven by the promoter of the *At5g17340 Arabidopsis* gene, a gene highly expressed in the male gametophyte. Strong GUS histochemical staining is detected in the uninucleate microspores. Strong GUS, and GFP, histological labelling is also detected in the subsequent stages of male gametophyte development. The potential applications of this marker line are discussed.

**Electronic supplementary material:**

The online version of this article (doi:10.1186/2193-1801-2-237) contains supplementary material, which is available to authorized users.

## Introduction

The *Arabidopsis* male gametophyte development is well characterised at the cytological level, and is a good system to study mitotic cell divisions. It is also a good system to indirectly characterise meiosis, since all four products of a single male meiotic cell division are viable, giving rise to microspores and pollen grains. The haploid unicellular uninucleate microspores undergo two rounds of mitotic divisions. The first mitotic cell division gives rise to bicellular pollen grains. As a result of the second mitotic cell division, tricellular pollen grains containing two gamete cells (the sperm cells) are formed. The bicellular pollen grain contains two *nuclei*, a condensed germinative *nucleus* and a large vegetative *nucleus*. The tricellular pollen contains a large vegetative *nucleus* and two condensed sperm *nuclei* (da Costa-Nunes and Grossniklaus 2003; Johnson-Brousseau and McCormick 2004; Borg et al. 2009). During pollination, the tricellular pollen grain germinates on the stigmatic *papillae*, producing a pollen tube that grows down the pistil and is attracted to one ovule (which holds the female gametophyte) (Shimizu and Okada 2000). While in wild-type Arabidopsis, the microspores and pollen grains are individualised, in the *quartet* (*qrt*) mutants the four male meiotic products remain bound together in a tetrahedron (tetrad) configuration throughout the whole of gametophyte development, even during pollination. Hence the *qrt* mutations allow the scrutiny of the fate of all four products of a single meiotic cell division (Preuss et al. 1994).

To characterise male gametophyte development and pollination, several transgenic marker lines exhibiting *ß-glucuronidase* induced (GUS) staining or green fluorescence protein (GFP) signal at different stages of the male gametophyte have been produced and used. One of the marker lines generally used in studies involving the male gametophyte, is the one carrying the *pLAT52*:*UidA* construct (Eady et al. 1995; Johnson et al. 2004). This construct, which contains a promoter from a tomato gene (*pLAT52*), does not induce GUS staining in all *Arabidopsis* male gametophyte stages. It only induces GUS staining immediately before the first gametophytic mitotic division, and in later stages of the male gametophyte (in bicellular and tricellular pollen grains) (Eady et al. 1994). Likewise, the *pLAT52:GFP* marker line has been reported to induce fluorescence signal in pollen grains, as well as in pollen tubes (Palanivelu and Preuss 2006; Francis et al. 2007). Another Arabidopsis marker line that uses a non-Arabidopsis promoter to induce strong and specific GUS staining in the male gametophyte, is the *pBnM3.4*:*UidA* marker line. In this marker line, the *Brassica napus* promoter sequence (*pBnM3.4*) induces GUS staining in *Arabidopsis* bicellular and tricellular pollen, yet, unlike *pLAT52*:*UidA*, it also induces strong GUS staining in *Arabidopsis* uninucleate microspores (Fourgoux-Nicol et al. 1999). Several *Arabidopsis* promoter sequences have also been shown to induce strong GUS staining in *Arabidopsis* flowers, in a male gametophyte and anther specific manner. However, these *Arabidopsis* promoters, either do not induce GUS staining in all stages of the male gametophyte, or if they do, they do not exhibit strong GUS staining at some of these stages (in particular in uninucleate microspores) (Moore et al. 1997; Li et al. 1998; Honys et al. 2006; Takeda et al. 2006; Gibalová et al. 2009; Phan et al. 2011).

Here is reported the production and characterisation of a novel marker line (*pAt5g17340:UidA:GFP*) that uses an *Arabidopsis* promoter sequence to generate strong GUS staining in uninucleate microspores, as well as strong GUS staining and GFP signal in the subsequent pollen development stages, and hence it is a potentially good marker line to quickly and easily tag all stages of the Arabidopsis male gametophyte, including pollination.

## Methods

### Plant material

*Arabidopsis thaliana* landrace Columbia-0 (Col) (N1093) and the *qrt* mutant (*qrt1-1*; Landsberg *erecta* landrace; N8050) (Copenhaver et al. 1998) were obtained from the Nottingham *Arabidopsis* Stock Centre. Controlled cross-pollination was used to combine the histological marker construct with the *qrt1-1* mutation.

Prior to germination, seeds were sterilised and imbibed in the dark at 4°C, for three to four days. Seeds were germinated in sterile Petri dishes containing solid germination medium (GM) (MS medium + Gamborg B5 vitamins, 1% sucrose; 0.8% micro-agar - Duchefa) or GM complemented with hygromycin. Alternatively, seeds were germinated in pots containing a sterilised commercial mix of turf, soil and fertiliser; pH 5.5 - 6.5. Seeds were germinated and plants grown in growth chambers with a cycle of 16 hours of light at 22°C alternating with 8 hours of darkness at 19°C.

### Cloning and plant transformation

A 2381 nucleotide base pairs (bp) PCR product containing the upstream region (henceforth promoter) of the *At5g17340* gene, a gene highly expressed in uninucleate microspores (Honys and Twell 2004), was PCR amplified using Bio-X-ACT-TAQ-LONG (Bio-X-ACT) polymerase, the primers ^5′^GTGTTTTTCAGAAGTCTATGAGCTCATTAG^3′^ and ^5′^CTCCATGGTTGGATTTTTTAGGAAACTTTTG^3′^, and wild-type Col-0 landrace genomic DNA as template; *Sac*I and *Nco*I restriction sites were incorporated in the primers sequences (underlined nucleotides). The blunt-end PCR products were dATP tailed and cloned into the pGEMT-Easy (invitrogene) vector. The cloned inserts were excised with *Sac*I and partial *Nco*I digest and cloned into the *Sac*I/*Nco*I double digested pCAMBIA1303 binary vector (http://www.cambia.org/daisy/cambia/585). Sequencing was used to verify the identity of the cloned inserts, and to certify that the insert was cloned in the correct reading frame.

Col-0 plants were transformed using the inflorescence dipping protocol (Clough and Bent 1998) and *Agrobacterium tumefaciens* (GV3101 strain) carrying the plasmid pMP90RK (Koncz and Schell 1986) and the binary vector construct. Transformants were selected on GM supplemented with hygromycin. Transformants (carrying the *At5g17340* promoter construct) were genotyped using the GUSOUT1 (^5′^GACTTCGCGCTGATACCAGACG^3′^) and P5 (^5′^CATGCATAAGATATCGATATCAG^3′^) primers; plant genomic DNA was extracted using the Edwards et al. (1991) protocol. Transformed Columbia-0 landrace plants were cross-pollinated with the *qrt1-1* mutant (Landsberg *erecta* landrace) (Copenhaver et al. 1998). *qrt1-1*/*qrt1-1* plants carrying the construct were identified on a screen for pollen with the *qrt* phenotype, on hygromycin resistant plants.

### Histochemical GUS staining and microscopy

Histochemical GUS staining of inflorescences was carried out as described in Jefferson et al. (1987) and Rodrigues-Pousada et al. (1993), with over-night incubation, at 37°C, with X-Gluc (5-bromo-4-chloro-3-indolyl beta-3-D-glucuronic acid, cyclohexylammonium salt, MW = 520.8) substract solution (2 mg/ml X-Guc; 50 mM Na Phosphate buffer, pH 7.0; 2 mM K_3_Fe(CN)_6_; 2 mM K_4_Fe(CN)_6_; 10 mM EDTA; 0.1% Triton-X). After GUS staining incubation, the inflorescences were subjected to three consecutive ethanol washes (95%, 80%, 70% ethanol). Intact anthers and dissected anthers from GUS stained and non-GUS stained flower buds were prepared for microscopy observation and mounted in a solution containing 2 μg/ml of DAPI (4′,6-diamino-2-phenylindole, dihydrochloride). GFP was observed in freshly harvested microspores and pollen grains, or whole anthers, mounted in DAPI (2 μg/ml) solution. Observations were carried out in an epifluorescence and optical microscope (Zeiss Axioskop 2) and in a dissecting scope (Wild M3Z Magnifying scope); photographs were taken with an Axiocam (Zeiss) digital camera. Images were processed using the Photoshop and Microsoft Windows Paint programs.

## Results

To produce and obtain marker lines with strong and specific histological staining during the entire male gametophyte development, and in particular in the uninucleate microspores, promoter sequences of genes highly expressed in microspores were selected to drive the expression of the *UidA* and *GFP* markers genes. These two histological markers (GUS and GFP) were chosen because with these, simple and low cost male gametophyte specific histological labelling can be achieved and easily detected. The selection of the candidate genes (and hence, their promoters) was based on the data available from publications reporting gene expression in pollen, and other floral organs and somatic tissues (Honys and Twell 2004; Schmid et al. 2005; Winter et al. 2007).

### The *At5g17340* promoter sequence promotes strong GUS staining in anthers

One of the selected genes, the *At5g17340*, is a gene predicted to code for a plant specific 160 aminoacids transmembrane peptide. *At5g17340* is the gene with the highest expression level in uninucleate microspores; it is also highly expressed in bicellular and tricellular pollen (Honys and Twell 2004). Its expression is reported to be restricted to the male gametophyte and anthers; it is not expressed in female gametophyte cells (synergids, egg cell, central cell) and it has no significant expression in other flower components (petals, sepals) (Schmid et al. 2005; Winter et al. 2007; Wuest et al. 2010). It is also not expressed during meiosis (Yang et al. 2011).

**Figure 1:**
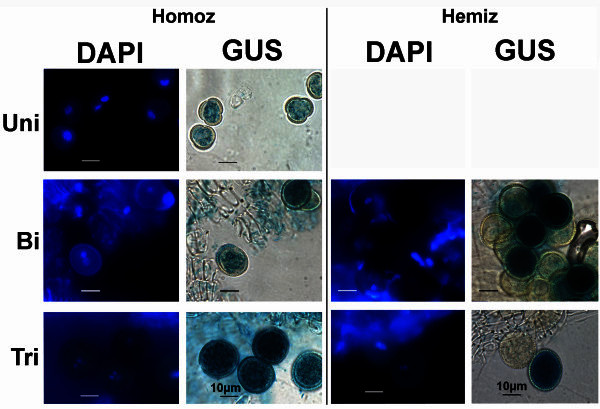
**Strong GUS staining in uninucleate microspores and pollen grains of the*pAt5g17340:UidA:GFP*marker line.** Strong GUS staining is detected in late uninucleate microspores (Uni), as well as in bicellular (Bi) and tricellular pollen (Tri) of plants homozygous for the *pAt5g17340:UidA:GFP* construct. Segregation of the marker gene (GUS stained and non-GUS stained pollen in a 1:1 ratio), as illustrated in the bicellular (Figure 1-Bi-Hemiz) and tricellular (Figure 1-Tri-Hemiz) pollen grains, indicates that the hemizygous plant has a single *UidA* marker gene insertion in the genome. Plates show DAPI stained *nuclei* (left column) of the GUS stained (right column) microspores and pollen grains in homozygous (Homoz) and hemizygous (Hemiz) plants. Uni- uninucleate microspore; Bi- bicellular pollen; Tri- tricellular pollen. Homozygous marker line plant: G63; Hemizygous marker line plant: GC3. GUS staining obtained after overnight incubation. Scale bars correspond to 10 μm.

The amplified 2353 bp promoter sequence of the *At5g17340* gene (*pAt5g17340*) was cloned in the pCAMBIA1303 binary vector to promote the simultaneous expression of the marker genes *UidA* (to induce GUS staining) and *GFP*. Transformation of Col-0 plants with the binary vector carrying the *pAt5g17340:UidA:GFP* construct, yielded 26 different independently transformed plants, 22 of which induce GUS staining in pollen. 15 of these lines induce GUS staining specifically in anthers and the male gametophyte. The GUS staining screen, focusing only on the flowers of these 15 lines, showed that GUS staining is detected in anthers (Additional file 1: Figure S1), in the *tapetum* (not shown) and the male gametophyte (Figure 1). It also showed that the anther specific GUS staining is developmentally dependent; it is absent in younger (smaller) flowers and it is present in anthers of older (bigger) flowers (Additional file 1: Figure S1b-e). 6 out of the 15 screened lines, also strongly GUS stain petals, sepals and carpels, after overnight incubation with X-Gluc (data not shown). Some of the lines with an anther specific GUS staining pattern also weakly stain the *papillae*. No GUS staining is detected in wild-type inflorescence after over-night incubation with X-Gluc (Additional file 1: Figure S1a).

### Strong GUS staining is detected in the male gametophyte, from the uninucleate microspore stage to pollen tube germination

**Figure 2:**
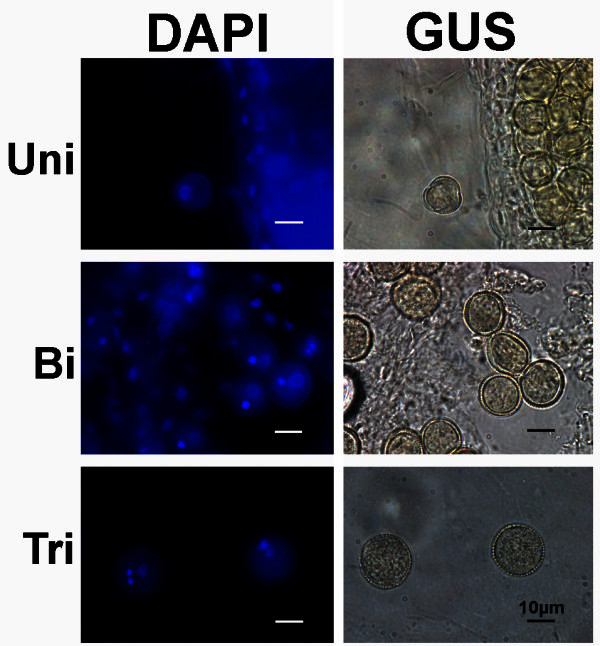
**X-Gluc incubated Col-0 uninucleate microspores and pollen grains.** No GUS staining (right column) is detected in late uninucleate microspores (Uni), nor in the bicellular (Bi) or tricellular (Tri) pollen grains of wild-type Col-0 landrace. The inflorescences were incubated overnight with X-Gluc substract to induce GUS staining. Plates show DAPI stained *nuclei* (left column) of the X-Gluc incubated (right column) microspores and pollen grains. Uni- uninucleate microspore; Bi- bicellular pollen; Tri- tricellular pollen. Col-0 plant. Scale bars correspond to 10 μm.

Further characterisation of the marker line was carried out in 8 of the plant lines exhibiting strong anther specific GUS staining. Very weak GUS staining can be first detected in microspores at the early microspore stage (early uninucleate microspore) (Additional file 2: Figure S2a). No GUS staining was detected in flowers at earlier developmental stages (data not shown). Later in development, strong GUS staining is detected in uninucleate microspores (late uninucleate microspores) (Figure 1-Uni; Additional file 2: Figure S2b), as well as in bicellular (Figure 1-Bi) and tricellular pollen grains (Figure 1-Tri). In lines homozygous (Homoz) for the marker gene, all pollen grains are GUS stained (Figure 1-Homoz); hemizygous (Hemiz) plants contain GUS stained and non-GUS stained pollen grains, and exhibit a 1:1 segregation ratio of the marker gene in the male gametophyte, as illustrated in bicellular and tricellular pollen grains (Figure 1-Bi-Hemiz and 1-Tri-Hemiz). Col-0 flowers incubated with X-Gluc do not exhibit any GUS staining in the male gametophyte (Figure 2).

**Figure 3:**
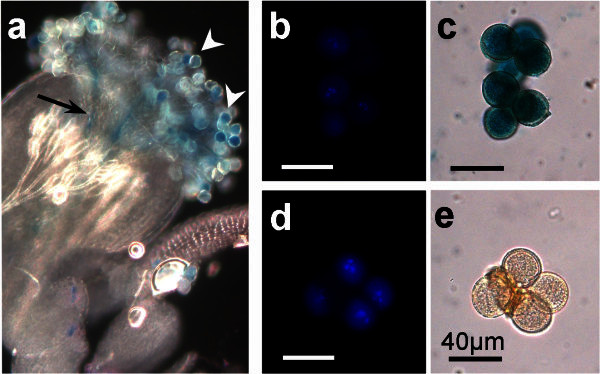
***pAt5g17340:UidA:GFP*induced GUS staining in*qrt*mutant pollen and during pollination.** GUS staining is also detected in the germinating pollen grains (white arrowheads) and in the germinated pollen tube inside the style (black arrow) (**a**). GUS stained tetrads of tricellular pollen grains from the *pAt5g17340:UidA:GFP* / *qrt1-1*/*qrt1-1* marker line (**c**) and tetrad of *qrt1-1*/*qrt1-1* mutant tricellular pollen grains carrying no marker gene (**e**). DAPI staining (**b**, **d**) of the X-Gluc incubated pollen grains (**c**, **e**). GUS staining obtained after overnight incubation. Marker line plants: *qrt1-1* and GC30 (with *qrt1-1* pollen); G57 (pollination). Scale bars correspond to 40 μm.

GUS staining, in the marker line, is also detected in germinating pollen grains attached to the stigmatic *papillae* (Figure 3a, white arrowheads) and in the pollen tubes growing down the stigma (Figure 3a, black arrow).

Selected plants of the *pAt5g17340:UidA:GFP* marker line were crossed to the *qrt1-1* mutant (Copenhaver et al. 1998). In this new lines (*pAt5g17340:UidA:GFP* / *qrt1-1*/*qrt1-1*), strong GUS staining is detected in the tetrads of pollen grains (Figure 3b, 3c), in contrast with the lack of GUS staining (after incubation with X-Gluc substract) in the tetrad of pollen grains of a *qrt1-1* mutant lacking the marker gene (Figure 3d, 3e). This *pAt5g17340:UidA:GFP* / *qrt1-1*/*qrt1-1* line was produced with the aim of aiding the characterisation of mutations affecting the male gametophyte or male meiosis.

### GFP signal is easily detected in bicellular and tricellular pollen grains

**Figure 4:**
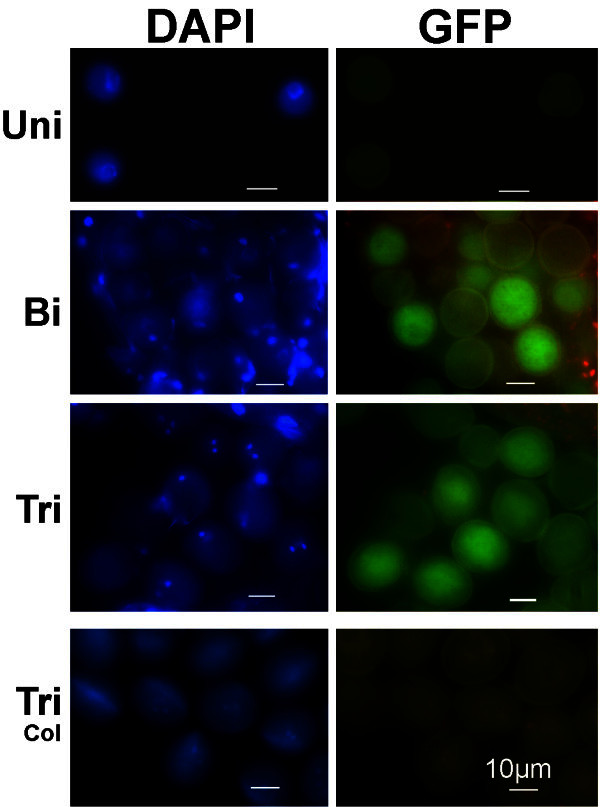
**GFP labelling in pollen grains of the*pAt5g17340:UidA:GFP*marker line.** GFP signal is clearly detected in bicellular (Bi) and tricellular (Tri) pollen grains of the *pAt5g17340:UidA:GFP* marker line. At the late uninucleate microspore stage (Uni), the *pAt5g17340:UidA:GFP* marker line GFP signal is very weak or undetectable. Segregation of the GFP signal (GFP *versus* non GFP - 1:1 ratio) is detected in the bicellular (Bi) and tricellular (Tri) pollen of a plant hemizygous for the *GFP* marker gene, indicating a single marker gene insertion in the genome. No GFP signal is detected in Col-0 tricellular pollen grains (Tri-Col). Plates show DAPI stained *nuclei* (left column) of the microspores and pollen grains shown in the right column. Uni- uninucleate microspore; Bi- bicellular pollen; Tri- tricellular pollen; Tri-Col- wild-type Col-0 tricellular pollen. Marker line plant: G65. Scale bars correspond to 10 μm.

No unambiguous GFP fluorescence signal is detected in uninucleate microspores (Figure 4-Uni) of the *pAt5g17340:UidA:GFP* marker line, in contrast with the strong GUS staining detected at this stage (Figure 1-Uni). In bicellular and tricellular pollen, however, strong GFP signal (Figure 4-Bi and 4-Tri) is detected; a signal that is clearly stronger than the pollen auto-fluorescence (Johnson-Brousseau and McCormick 2004). The segregation of the GFP marker gene is also clearly and easily detected, in a 1:1 ratio (GFP:no-GFP), in the gametophyte of hemizygous plants (Figure 4-Bi, 4-Tri). This same GFP pattern (in the male gametophyte) is detected in this line (G65) as well as in others (GC7 - data not shown).

The construct that induces GFP expression in this line (G65; Figure 4), also induces strong anther specific and developmentally regulated GUS staining in the G138 plants (plants obtained from G65 self-pollination; Additional file 1: Figure S1). Hence, the same line can be used for both GUS staining and GFP labeling of the male gametophyte. At least, two more independent lines of the *pAt5g17340:UidA:GFP* marker line have been shown to provide both GFP labeling and GUS staining (data not shown).

Figure 4-Tri-Col shows wild-type (Col-0) tricellular pollen grains lacking GFP fluorescence signal.

Further information concerning the plant lines described in this report is provided in the Additional file 3: Table S1.

## Discussion

The aim of the work reported here was to obtain marker lines that induce strong histological labelling (GUS or GFP) in all stages of the male gametophyte. The ultimate goal would be to use these lines in the characterisation of the male gametophyte of male-fertility-impaired mutants. Since many of these mutants produce aborted gametophytic cells (Johnson et al. 2004; Takeda et al. 2006; da Costa-Nunes and Viegas 2009; Gibalová et al. 2009), it is important to induce GUS staining before they collapse, otherwise it becomes difficult or impossible to detect GUS staining in these mutant gametophytic cells (Johnson et al. 2004). Therefore, the *pAt5g17340:UidA:GFP* marker line was produced to strongly label the male gametophyte, as early as possible, in uninucleate microspores (Figure 1-Uni), as well as the subsequent stages of male gametophyte development.

### *pAt5g17340:UidA:GFP* is a good marker line to induce strong male gametophyte specific GUS staining, in uninucleate microspores and pollen grains

In this report, it is shown that the flowers from the new *pAt5g17340:UidA:GFP* marker line plants exhibit strong and anther specific GUS staining (Additional file 1: Figure S1b-e), in uninucleate microspores and bicellular and tricellular pollen grains, after incubation with X-Gluc (Figure 1; Additional file 2: Figure S2). GUS staining is first detected in anthers accommodating uninucleate microspores (Additional file 2: Figure S2a), which indicates that the *pAt5g17340:UidA:GFP* construct does not induce GUS staining in anthers of flowers at the meiotic and pre-meiotic stage.

Of the several reported marker lines that use an *Arabidopsis* gene promoter to induce strong GUS staining in the male gametophyte, *pAt3g45150:UidA* is the only marker line that has been shown to induce particularly strong GUS staining in uninucleate microspores (Takeda et al. 2006). The intensity of the reported GUS staining is comparable to the one detected in the uninucleate microspores of the *pAt5g17340:UidA:GFP* marker line (Figure 1-Uni). Yet, unlike *pAt5g17340:UidA:GFP*, the *pAt3g45150:UidA* does not induce GUS staining at the last stages of pollen development (Takeda et al. 2006).

Like *pAt5g17340:UidA:GFP,* there are other marker lines that use *Arabidopsis* promoters to induce convincing GUS staining in all the male gametophytic stages (Honys et al. 2006), yet the reported staining intensity in their uninucleate microspores is not as strong as in the *pAt5g17340:UidA:GFP* marker line.

In conclusion, the GUS staining data indicates that the novel marker line (*pAt5g17340:UidA:GFP*) is a good tool to characterise all stages of male gametophyte development. The *pAt5g17340:UidA:GFP* marker line is particularly good to characterise uninucleate microspores (Figure 1), and it can also be used to characterise pollen tube germination and pollination (Figure 3a). Furthermore, the data indicates that the GUS staining intensity and pattern observed in the *pAt5g17340:UidA:GFP* marker line (Figure 1) is coherent with the *At5g17340* reported male gametophyte gene expression microarray data (Honys and Twell 2004) that indicates that this gene is expressed in high levels in uninucleate microspores, bicellular and tricellular pollen grains.

### The promoter sequences of *At5g17340* and its *Brassica napus* homologous gene share the ability to induce strong histochemical staining during the entire male gametophyte

There is one other *Arabidopsis* marker line, that like the *pAt5g17340:UidA:GFP* marker line, has been reported to induce strong GUS staining in uninucleate microspores, and in bicellular and tricellular pollen grains (Fourgoux-Nicol et al. 1999). In this *Arabidopsis* marker line (*pBnM3.4:UidA*), the promoter (*pBnM3.4*) of the *BnM3.4*/CL1Contig2422 *Brassica napus* gene is used to induce strong anther specific GUS staining. *BnM3.4* and *At5g17340* are homologous genes (Fourgoux-Nicol et al. 1999; Whittle et al. 2010). Both genes are expressed at high levels; *BnM3.4* is the *Brassica napus* gene with the third highest expression level in *Brassica napus* microspores and early bicellular pollen (Whittle et al. 2010) while *At5g17340* is the gene with the highest expression level in *Arabidopsis* uninucleate microspores (Honys and Twell 2004). The strong GUS staining obtained in the *pAt5g17340:UidA:GFP* and *pBnM3.4:UidA* marker lines is consistent with these high levels of gene expression. The GUS (or GFP) staining pattern also indicates, in particular in hemizygous plants segregating GUS stained and non-GUS stained (or GFP) pollen grains (Figures 1-Bi-Hemiz, 1-Tri-Hemiz, 4-Bi and 4-Tri) (Fourgoux-Nicol et al. 1999), that these homologous genes are gametophytically expressed. Hence, the promoters of the *At5g17340* and *BnM3.4* homologous genes share and conserve the ability to induce high levels of gene expression (particularly in uninucleate microspores), male gametophytic specific gene expression, and similar and strong GUS staining patterns.

### *pAt5g17340:UidA:GFP* induced strong GFP fluorescence is suitable to characterise bicellular and tricellular pollen grains

GFP signal (Figure 4) is clearly detected in plants carrying the *pAt5g17340:UidA:GFP* construct in both bicellular and tricellular pollen grains, yet no clear positive GFP fluorescence signal was observed in uninucleate microspores (Figure 4-Uni); It is not known why the GFP signal is not detected, with epifluorescence microscopes, in uninucleate microspores of this marker line. Hence, this marker line, like other previously reported marker lines such as the *pLAT52:GFP* (Francis et al. 2007), is only suitable to characterise (with GFP) the later stages of pollen development.

### The *pAt5g17340:UidA:GFP* / *qrt1-1*/*qrt1-1* marker line

In this line, that combines the *pAt5g17340:UidA:GFP* construct with the *qrt1-1* mutation, the products of male meiosis are held together during the entire male gametophyte development. This line was produced to facilitate the characterisation and scrutiny of the fate, at all stages of male gametophyte development, of all meiotic products obtained from each single abnormal mutant meiotic cell division as well as of the microspores and pollen grains formed in gametophytic mutants. The strong GUS staining in uninucleate microspores (Figure 1-Uni), combined with the *qrt1-1* mutation (Figure 3c), has potential interest for the characterisation of meiotic mutants, or gametophytic mutants, that produce aborted and collapsed gametophytic cells at the early stages of male gametophyte development.

## Electronic supplementary material

Additional file 1: Figure S1: GUS staining in the inflorescence is anther specific and developmentally regulated. Inflorescence side view (a, b, d, e) and top view (c) showing GUS staining only in the anthers of the older (outer) flowers of different independently obtained transformed lines (b, c, d, e) of the *pAt5g17340:UidA:GFP* marker line. No GUS staining is detected in the inner (younger) flowers of the inflorescence from different independent lines from this marker line (b, c, d, e) nor in the wild-type Col-0 inflorescence (a). White arrow points to the smaller (younger) flower buds where GUS staining is first detected (e). Marker line plants: GC21 (b, c); G138 (d) and G57 (e) are descendent from three different independently transformed lines; line G138 is descendent from the self-pollinated G65 line (see Figure 4); self-pollination of G57 (see also Additional file 2: Figure S2) gave origin to the line G63 (see Figure 1). Col-0 (a). GUS staining obtained after overnight incubation. (PDF 164 KB)

Additional file 2: Figure S2: GUS staining in flowers at different developmental stages, and the respective uninucleate microspores and pollen grains. All flowers were harvested from a single inflorescence of a *pAt5g17340:UidA:GFP* marker line plant. a, b - Young flowers with GUS stained anthers, containing uninucleate microspores; a - early uninucleate microspores with weak GUS staining; b - late uninucleate microspore with strong GUS staining. Flowers smaller than the one depicted in plate ‘a’ did not exhibit GUS staining (not shown). c, d, e, f - Flowers with anthers containing GUS stained bicellular (c, d) and tricellular (e) pollen grains. Neither DAPI nor GUS stained pollen grains from the flower ‘f’ are shown. GUS stained pollen tube elongating in the pistil is detected in plate ‘e’ (Flower). f - An older flower with GUS stained pollen grains attached to the stigma’s *papillae* and inside the dehiscent anthers. The microspores and pollen grains that are shown were dissected from one or two anthers removed from the flowers depicted in the (Flower) plates. Flower plates - Whole flower with GUS stained anthers. DAPI plates show the DAPI stained *nuclei* of the GUS stained microspores and pollen grains (GUS plates). Marker line plant: G57. GUS staining obtained after overnight incubation. Scale bars correspond to 0.4 mm in Flower plates, and to 15 μm in DAPI and GUS plates. (PDF 309 KB)

Additional file 3: Table S1: The different (and independently transformed) lines from the *pAt5g17340:UidA:GFP* marker line reported in this manuscript. Table listing the lines reported in this manuscript. (PDF 16 KB)
