# Supplementary material for: A novel Arabidopsis marker line that strongly labels uninucleate microspores and the subsequent male gametophyte development stages
Source: Springerplus. 2013 May 24;2:237. doi: 10.1186/2193-1801-2-237 (PMC3671114; doi:10.1186/2193-1801-2-237)
Supplement: Supplementary file 1 — Additional file 1: Figure S1: GUS staining in the inflorescence is anther specific and developmentally regulated. Inflorescence side view (a, b, d, e) and top view (c) showing GUS staining only in the anthers of the older (outer) flowers of different independently obtained transformed lines (b, c, d, e) of the pAt5g17340:UidA:GFP marker line. No GUS staining is detected in the inner (younger) flowers of the inflorescence from different independent lines from this marker line (b, c, d, e) nor in the wild-type Col-0 inflorescence (a). White arrow points to the smaller (younger) flower buds where GUS staining is first detected (e). Marker line plants: GC21 (b, c); G138 (d) and G57 (e) are descendent from three different independently transformed lines; line G138 is descendent from the self-pollinated G65 line (see Figure 4); self-pollination of G57 (see also Additional file 2: Figure S2) gave origin to the line G63 (see Figure 1). Col-0 (a). GUS staining obtained after overnight incubation. (PDF 164 KB) [file 40064_2013_304_MOESM1_ESM.pdf]

Additional file 1: Figure S1

A novel *Arabidopsis* marker line that strongly labels uninucleate microspores and the subsequent male gametophyte development stages.

José António da Costa-Nunes[1*]

[*] Corresponding author
Email: j.dacostanunes@wolfson.oxon.org

[1] CBAA - Instituto Superior de Agronomia, Universidade Técnica de Lisboa, Tapada da Ajuda, Lisboa P-1349-017, Portugal

Additional file 1: Figure S1

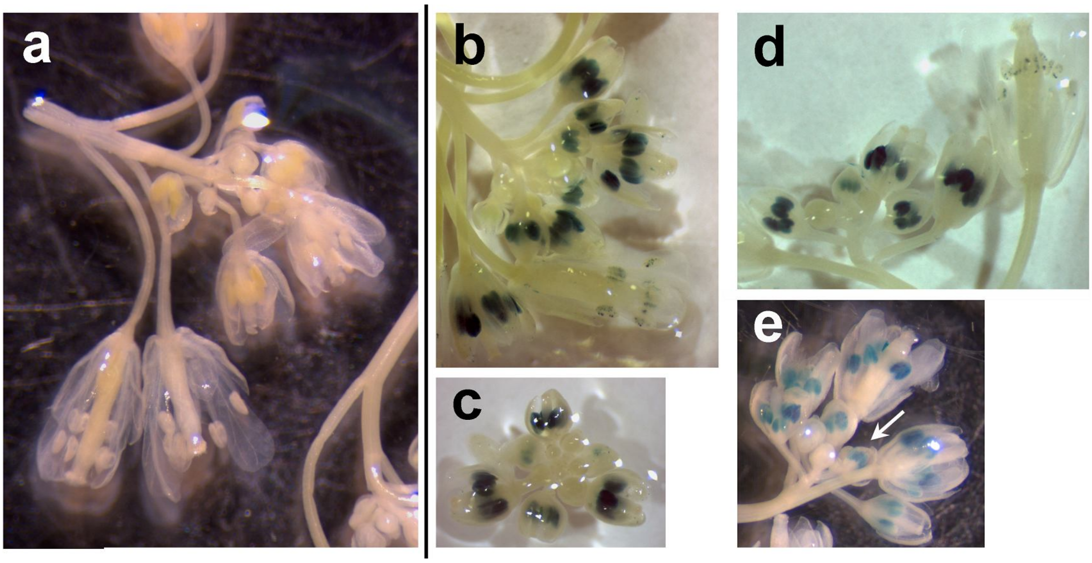

Additional file 1: Figure S1 - GUS staining in the inflorescence is anther specific and developmentally regulated.

Inflorescence side view (a, b, d, e) and top view (c) showing GUS staining only in the anthers of the older (outer) flowers of different independently obtained transformed lines (b, c, d, e) of the *pAt5g17340:UidA:GFP* marker line. No GUS staining is detected in the inner (younger) flowers of the inflorescence from different independent lines from this marker line (b, c, d, e) nor in the wild-type Col-0 inflorescence (a). White arrow points to the smaller (younger) flower buds where GUS staining is first detected (e). Marker line plants: GC21 (b, c); G138 (d) and G57 (e) are descendent from three different independently transformed lines; line G138 is descendent from the self-pollinated G65 line (see Figure 4); self-pollination of G57 (see also Additional file 2: Figure S2) gave origin to the line G63 (see Figure 1). Col-0 (a). GUS staining obtained after overnight incubation
