# Supplementary material for: A novel Arabidopsis marker line that strongly labels uninucleate microspores and the subsequent male gametophyte development stages
Source: Springerplus. 2013 May 24;2:237. doi: 10.1186/2193-1801-2-237 (PMC3671114; doi:10.1186/2193-1801-2-237)
Supplement: Supplementary file 2 — Additional file 2: Figure S2: GUS staining in flowers at different developmental stages, and the respective uninucleate microspores and pollen grains. All flowers were harvested from a single inflorescence of a pAt5g17340:UidA:GFP marker line plant. a, b - Young flowers with GUS stained anthers, containing uninucleate microspores; a - early uninucleate microspores with weak GUS staining; b - late uninucleate microspore with strong GUS staining. Flowers smaller than the one depicted in plate ‘a’ did not exhibit GUS staining (not shown). c, d, e, f - Flowers with anthers containing GUS stained bicellular (c, d) and tricellular (e) pollen grains. Neither DAPI nor GUS stained pollen grains from the flower ‘f’ are shown. GUS stained pollen tube elongating in the pistil is detected in plate ‘e’ (Flower). f - An older flower with GUS stained pollen grains attached to the stigma’s papillae and inside the dehiscent anthers. The microspores and pollen grains that are shown were dissected from one or two anthers removed from the flowers depicted in the (Flower) plates. Flower plates - Whole flower with GUS stained anthers. DAPI plates show the DAPI stained nuclei of the GUS stained microspores and pollen grains (GUS plates). Marker line plant: G57. GUS staining obtained after overnight incubation. Scale bars correspond to 0.4 mm in Flower plates, and to 15 μm in DAPI and GUS plates. (PDF 309 KB) [file 40064_2013_304_MOESM2_ESM.pdf]

Additional file 2: Figure S2

A novel *Arabidopsis* marker line that strongly labels uninucleate microspores and the subsequent male gametophyte development stages.

José António da Costa-Nunes[1*]

[*] Corresponding author
Email: j.dacostanunes@wolfson.oxon.org

[1] CBAA - Instituto Superior de Agronomia, Universidade Técnica de Lisboa, Tapada da Ajuda, Lisboa P-1349-017, Portugal

Additional file 2: Figure S2

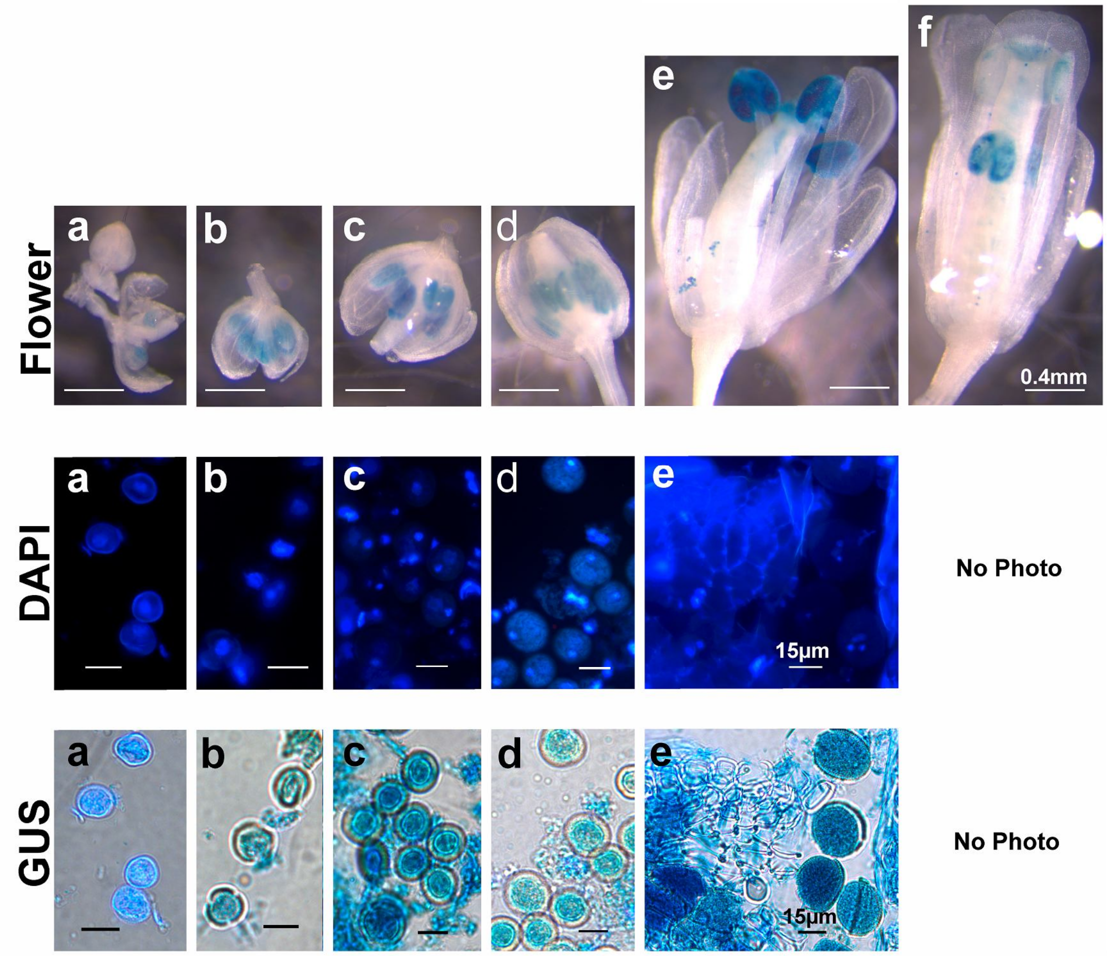

Additional file 2: Figure S2 - GUS staining in flowers at different developmental stages, and the respective uninucleate microspores and pollen grains.

All flowers were harvested from a single inflorescence of a *pAt5g17340:UidA:GFP* marker line plant.

a, b - Young flowers with GUS stained anthers, containing uninucleate microspores; a - early uninucleate microspores with weak GUS staining; b - late uninucleate microspore with strong GUS staining. Flowers smaller than the one depicted in plate 'a' did not exhibit GUS staining (not shown).

c, d, e, f - Flowers with anthers containing GUS stained bicellular (c, d) and tricellular (e) pollen grains. Neither DAPI nor GUS stained pollen grains from the flower 'f' are shown. GUS stained pollen tube elongating in the pistil is detected in plate 'e' (Flower). f - An older flower with GUS stained pollen grains attached to the stigma's *papillae* and inside the dehiscent anthers.

The microspores and pollen grains that are shown were dissected from one or two anthers removed from the flowers depicted in the (Flower) plates.

Flower plates - Whole flower with GUS stained anthers. DAPI plates show the DAPI stained *nuclei* of the GUS stained microspores and pollen grains (GUS plates).

Marker line plant: G57. GUS staining obtained after overnight incubation. Scale bars correspond to 0.4mm in Flower plates, and to 15µm in DAPI and GUS plates
