# Supplementary material for: A novel Arabidopsis marker line that strongly labels uninucleate microspores and the subsequent male gametophyte development stages
Source: Springerplus. 2013 May 24;2:237. doi: 10.1186/2193-1801-2-237 (PMC3671114; doi:10.1186/2193-1801-2-237)
Supplement: Supplementary file 3 — Additional file 3: Table S1: The different (and independently transformed) lines from the pAt5g17340:UidA:GFP marker line reported in this manuscript. Table listing the lines reported in this manuscript. (PDF 16 KB) [file 40064_2013_304_MOESM3_ESM.pdf]

Additional file 3: Table S1

A novel *Arabidopsis* marker line that strongly labels uninucleate microspores and the subsequent male gametophyte development stages.

José António da Costa-Nunes[1*]

[*] Corresponding author
Email: j.dacostanunes@wolfson.oxon.org

[1] CBAA - Instituto Superior de Agronomia, Universidade Técnica de Lisboa, Tapada da Ajuda, Lisboa P-1349-017, Portugal

Additional file 3: Table S1

The different (and independently transformed) lines from the *pAt5g17340:UidA:GFP* marker line reported in this manuscript.

Independent transformant / lines:	Marker line construct:	Genotype / background:	Notes:
**GC21** (*qrt1-1*)	*pAt5g17340:UidA:GFP*	F2 Col-0 x Landsberg	- Descendent from one (1st) of the independently transformed *pAt5g17340:UidA:GFP* lines and *qrt1-1*.<br>- Line containing plants homozygous and heterozygous for the *qrt1-1* mutation and homozygous and hemizygous for the *pAt5g17340:UidA:GFP* construct.
**G65**	*pAt5g17340:UidA:GFP*	Col-0	- Descendent from a 2nd independently transformed *pAt5g17340:UidA:GFP* line.<br>- Line containing plants homozygous and hemizygous for the *pAt5g17340:UidA:GFP* construct.<br>- The anthers of hemizygous G65 plants exhibit a 50% GFP labelled *versus* 50% non-GFP labelled pollen ratio; indication of a single T-DNA insertion.<br>- Lines descendent from G65, that are heterozygous for *qrt1-1* and hemizygous for the *pAt5g17340:UidA:GFP* construct (Hygromycin Resistant:Hygromycin sensitive; 3:1), are also available.
**G138**	*pAt5g17340:UidA:GFP*	Col-0	- Descendent from self-pollinated G65.<br>- Is homozygous for the *pAt5g17340:UidA:GFP* construct.

**G57**	*pAt5g17340:UidA:GFP*	Col-0	- Descendent from a 3rd independently transformed *pAt5g17340:UidA:GFP* line.<br><br>- Lines descendent from G57, that are heterozygous for *qrt1-1* and hemizygous for the *pAt5g17340:UidA:GFP* construct (Hygromycin Resistant:Hygromycin sensitive; 3:1), are also available.
**G63**	*pAt5g17340:UidA:GFP*	Col-0	- Descendent from self-pollinated G57.<br><br>- Line containing plants homozygous and hemizygous for the *pAt5g17340:UidA:GFP* construct.<br><br>- The anthers of hemizygous G63 plants exhibit a 50% GUS stained *versus* 50% non-GUS stained pollen ratio; indication of a single T-DNA insertion.
**GC3** (*qrt1-1*)	*pAt5g17340:UidA:GFP*	F1<br>Col-0 x Landsberg	- GC3 is descendent from G57 and *qrt1-1*.<br><br>- Line containing plants heterozygous for the *qrt1-1* mutation and hemizygous for the *pAt1g02790:UidA:GFP* construct.
**GC7** (*qrt1-1*)	*pAt5g17340:UidA:GFP*	F1<br>Col-0 x Landsberg	- Descendent from a 4th independently transformed *pAt5g17340:UidA:GFP* line and *qrt1-1*.<br><br>- Line containing plants heterozygous for the *qrt1-1* mutation and hemizygous for the *pAt1g02790:UidA:GFP* construct.
**GC30** (*qrt1-1*)	*pAt5g17340:UidA:GFP*	F2<br>Col-0 x Landsberg	- GC30 is descendent from self-pollinated GC7.<br><br>- Line containing plants homozygous and heterozygous for the *qrt1-1* mutation and homozygous and hemizygous for the *pAt5g17340:UidA:GFP* construct.

Additional file 3: Table S1 - Table listing the lines reported in this manuscript
